# Lotus seeds (*Nelumbinis semen*) as an emerging therapeutic seed: A comprehensive review

**DOI:** 10.1002/fsn3.2313

**Published:** 2021-05-06

**Authors:** Muzalfa Arooj, Saira Imran, Muhammad Inam‐ur‐Raheem, Muhammad Shahid Riaz Rajoka, Aysha Sameen, Rabia Siddique, Amna Sahar, Shiza Tariq, Ayesha Riaz, Abid Hussain, Azhari Siddeeg, Rana Muhammad Aadil

**Affiliations:** ^1^ National Institute of Food Science and Technology University of Agriculture Faisalabad Pakistan; ^2^ Food and Feed Immunology Group Graduate School of Agricultural Science Tohoku University Sendai Japan; ^3^ Department of Chemistry Government College University Faisalabad Pakistan; ^4^ Department of Food Engineering University of Agriculture Faisalabad Pakistan; ^5^ Institute of Home Sciences University of Agriculture Faisalabad Pakistan; ^6^ School of Food Science and Engineering South China University of Technology Guangzhou China; ^7^ Department of Food Engineering and Technology Faculty of Engineering and Technology University of Gezira Wad Medani Sudan

**Keywords:** bioactive compounds, extraction techniques, functional foods, lotus seeds, polyphenols, therapeutic potential

## Abstract

*Nelumbinis semen* is commonly known as lotus seeds that have been used as a vegetable, functional food, and medicine for 7,000 years. These are low caloric, a rich source of multiple nutrients and bioactive constituents, which make it a unique therapeutic food. *N*. *semen* plays an important part in the physiological functions of the body. Nowadays, people are more conscious about their health and desire to treat disease naturally with minimal side effects. So, functional foods are getting popularity due to a wide range of essential constituents, which are associated to decrease the risk of chronic diseases. These bioactive compounds from seeds are involved in anti‐adipogenic, antioxidant, antitumor, cardiovascular, hepato‐protective, anti‐inflammatory, anti‐fertility, anti‐microbial, anti‐viral, hypoglycemic, etc. Moreover, the relationship between functional compounds along with their mechanism of action in the body, their extraction from the seeds for further research would be of great interest.

## Graphical abstract

Lotus seeds contain many bioactive compounds like alkaloid, flavonoids etc.
Lotus seeds exert anti‐inflammatory, anti‐cancer, hypoglycemic activity and others.
Lotus seeds have beneficial aspects by decreasing the risk of chronic diseases.
Relationship between bioactive compounds and their mechanism of action in the body.

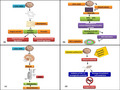

## 1 INTRODUCTION

Functional foods are like conventional foods, which people use in the normal routine and the only difference is the provision of health benefits. These foods control diseases at different stages and prevent their further progression (Abuajah et al., 2015). In general, both functional foods and lifestyle changes can have a great positive impact on the well‐being and the health of humans. Due to the presence of a wide range of phytoconstituents and bioactive compounds, functional foods have gained massive popularity in the area of nutritional sciences (Perveen et al., 2015; Shahzad et al., 2021). These compounds activate and deactivate body mechanisms like triggering body immunity, modulating, and detoxification. These also play an important role in non‐communicable diseases, inflammatory disorders, insomnia, memory‐related issues, and various type of degenerative age‐related diseases (Giampieri et al., 2014). *Nelumbo* *nucifera*, is a plant of the monogeneric family Nymphaeaceae, an aquatic perennial herb in nature, having an elegant fragrance and a sign of beauty and prosperity (Li et al., 2020; Yu et al., 2013). Globally, *N. lutea* and *N. nucifera* are the only two species of *N. nucifera*, which are found in Asia, Australia, eastern and southern North America (Islam et al., 2020). Seeds of lotus consist of three sections that is, seed epicarp, cotyledons, and embryo and all of these are well‐known due to their functional food properties and nutritional importance (Mukherjee et al., 2009). Commercially, there are two types of dried lotus seeds, which can be found as white and brown peel (Acharya & Srikanth, 2014). Radiocarbon dating showed that seeds of lotus have a maximum viability period of about 1,300 years (Ferrer‐Gallego et al., 2015). Lotus seeds consumed in many forms as raw or cooked, ripened, or un‐ripened, and mostly used in desserts (Chen et al., 2019). These seeds can also be used as roasted/ground, raw, and boiled into syrups (Moro et al., 2013). Lotus seed contains a diverse range of phytochemicals including alkaloids, flavonoids, polysaccharides, essential oils, glycosides, polyphenols, triterpenoids, etc., which have a wide range of pharmaceutical properties (Chen et al.,^2012^, ^2019^; Huang et al., 2009; Zhenjia et al., 2010). Studies on phytochemical properties of different parts of the lotus have revealed great therapeutic potentials. The pharmacological activities of lotus seeds on human health include antioxidant (Sujitha et al., 2013), antitumor (Menéndez‐Perdomo & Facchini, 2020), analgesic (Rajput et al., 2019), anti‐obesity (Sim et al., 2019), anti‐inflammatory (Harishkumar et al., 2020), analgesic (Rajput et al., 2019), anti‐obesity (Sim et al., 2019), cardiovascular, hepato‐protective, immune regulatory, improving memory, hypoglycemia and anti‐viral activities as well as for treating leprosy, halitosis, menorrhagia and fever (Zaidi & Srivastava, 2019). The lotus seeds are used for treating some ailments like chronic diarrhea insomnia, palpitations, poor digestion, enteritis, and cancer (Buddhadev & Buddhadev, 2014). According to the ‘Ministry of Health of the People's Republic of China’ lotus, seeds have been approved as “both foods as well as medicine” (Zhang et al., 2015). So, this review is based on the therapeutic effects of nutritional compounds of lotus seed, their mechanisms of action. This review would help to know the specific process needed to be up‐regulated or down‐regulated to mitigate that specific pathogenic condition. Although, the benefits of the mechanism studied can be summarized in two main categories: 1) development of essential knowledge and 2) precision of disease occurrences. The researchers had tried to explain the mechanism, but there is a need to further work on these mechanisms to get a clearer picture of the therapeutic potentials of lotus seeds.

## 2 GLOBAL PRODUCTION OF LOTUS SEEDS

The plant was anciently found in Asia, Iran, and Egypt and used as a metaphor for almost 4,000 years. Lotus is usually found along river Nile's bank (Kandeler & Ullrich, 2009). The plant was carried from Egypt to the Middle East ancient empires (Assyria). It was planted in China, Persia, and India as architectural motifs. It was then introduced from China to Japan, cultivated for thousands of years and now found in every botanical garden. For 7,000 years, lotus seeds have been grown as a vegetable as well as for medicinal purposes (Ming et al., 2013). Nowadays, it is an important plant from an economical point of view in countries of Asia. Lotus seeds are extensively cultivated in India, Japan, and China (Zhang et al., 2012). China being the tremendous cultivator and consumer all over the sphere, it was 0.2 million hectares (Ha) area for cultivation of lotus rhizome. The annual harvest of dry seeds was 15,000 tons in 1999. By 2003, the area used for cultivation was about 67, 300 hectares. According to the latest research, China produces 45,000 tons of dry seeds and 9 million rhizomes in 0.5–0.7 million hectares of area per year. It served as an industrial crop over an area of 40,000 Hectares (Guo, 2009; Liu et al., 2006). By 2012, the area for cultivation of Lotus in China was more than 100,000 Hectares (per year) while in 2014, the annual production of lotus seeds was about 1.2 × 10^8^ kg (Luo et al., 2016). By 2017, the production of lotus seeds reached 12,205 tons per year in Fujian Province corresponding to a GDP of about 1.8 billion Chinese Yuan (He et al., 2021). Lotus is a national flower of India, grows at both high and low altitudes. The production of seeds is about 200 to 250 kg/Ha and is sold as snacks in some places at a price of US $1.35/kg. Internationally its price is approximately US $200/1000 seeds. As these seeds are consumed in very little amount, their price may vary yearly (Bhat & Sridhar, 2008; Goel et al., 2001).

## 3 LOTUS SEEDS AND THEIR NUTRITIONAL PROFILE

Lotus seeds are rich in nutrients, but their content may vary due to differences in cultivation, environment, and varieties. However, physiological characteristics and efficacy of nutrients remain the same (Wang & Zhang, 2010). Lotus seeds not only contain macronutrients (proteins, carbohydrates, and fats) but also a particularly enormous quantity of minerals, such as phosphorus (P), calcium (Ca), magnesium (Mg), iron (Fe), and some other vitamins (Wu et al., 2007). The fresh lotus seeds were found to have 31.24 mg/ kg of vitamin C, which is an antioxidant and thus has stress coping and improving immunity abilities (Chouaibi et al., 2012). These seeds are not only rich in amino acid content and unsaturated fatty acids but also have a considerable amount of polysaccharides, superoxide dismutase (SOD), polyphenols, and other bioactive components (Bhat & Sridhar, 2008). Lotus seeds are rich in high‐quality essential amino acids with a heaven‐sent E/T ratio (essential amino acids/total amino acid) as recommended by WHO for the ideal protein source of 36% (Cai et al., 2011; Joint & Organization, 1973). The total calories provided by lotus seeds are 211.18 kcal/100g. It is therefore used as food in daily routine and for medicinal purpose in diseased condition (Musa et al., 2012). The nutritional composition of lotus seeds is given in Table 1.

**TABLE 1:** The nutritional profile of lotus seeds

Constituents	% dry basis	References
Moisture	9.10	(Chouaibi et al., 2012; Musa et al., 2012)
Protein	13.32	
Fat	2.82	
Fiber	20.27	
	Mg/100g	
Na	11.83	
K	191.67	
Ca	127.82	
Mg	10.86	
Cr	3.65	
Ni	11.02	
Fe	16.29	
Zn	8.78	
Vit C	31.24	

## 4 CHEMICAL CONSTITUENTS

Lotus seeds have a variety of chemical components, including flavonoids, glycosides, phenolic compounds, and alkaloids. Different constituents are scattered in different tissues of lotus, as the flavonoids are abundantly present in leaves, flowers, and plumule while the alkaloids are present in leaves and seeds (Chen et al., 2012; Huang et al., 2009; Kashiwada et al., 2005; Zhenjia et al., 2010). A detailed description of the reported main constituents in lotus seeds is given in Table 2.

**TABLE 2:** The chemical constituent in lotus seeds

Compounds	Components	References
Flavonoids and glycosides	Kampferol and glycosides, Astragalin, kaempferol 3‐O‐rob, kaempferol 3‐O‐deoxyhexose‐hexose, kaempferol 7‐O‐glu, Quercetin and glycosides, Isoquercitrin, hyperoside, quercetin 3‐O‐hexose, rutin, quercetin 3‐O‐deoxyhexose‐hexose, Rhamnetin and glycoside, isorhamnetin 3‐O‐hexose, isorhamnetin 3‐O‐deoxyhexose‐hexose, Myricetin and glycosides, myricetin 3‐O‐hexose, Lutelin and glycosides, luteolin7‐O‐neo, orientin, isoorientin, luteolin 6‐C‐glu−8‐C‐ara, Apigenin and glycosides, Isovitexin, apigenin 6‐C‐glu−8‐C‐xyl, vitexin, schaftoside, isoschaftoside, vicenin−2, apigenin 6‐C‐glu−8‐C‐rha, Tanins procyanidin (dimer)	(Chen et al., 2012; Kredy et al., 2010; Li et al., 2014; Ling et al., 2005; Liu, Zhu, Zhang, & Guo, 2017; Zhu et al., 2017)
Alkaloids	Aporphine, Nuciferine, Benzyl isoquinolines, 40‐methyl coclaurine plumules, 40‐methyl‐*N*‐methylcoclaurine, higenamine, higenamine 4‐O‐glu, Bisbenzylisoquinolines, Nelumboferine, Heterocycle, Methylcorypallines	(Itoh et al., 2011; Nakamura et al., 2013; Sharma et al., 2017)
Other secondary components	Monosacchrides, D‐arabinose, D‐glucose, D‐galactose, D‐mannose, L‐rhamnose, D‐lyxose, D‐glucuronic acid, L‐arabinose, Essential oils, 3‐carene, camphene, a‐pinene, 1–8‐cineole, linalool, geraniol, c‐gurjunene, s‐cadinol, Organic acid, Anisic acid, Steroids and sapogenins, Campesterol, isofucosterol, dehydrolanosterol, lanosterol, Saturated and unsaturated acids, Myristic acid, Monoglycrieds, 1‐palmitoyl, 2‐behenoyl glycerol, 1‐linoleoyl glycerol, 2‐Palmitoyl glycerol, 1‐oleoyl glycerol.	(Gao & Chen, 2003; Kim, Zhao, Shen, & Chang, 2013; Khan et al., 2016)
Phenolic acid	Protocatechuic acid, Caffeic acid, ellagic acid.	(Lin et al., 2018)

## 5 EXTRACTION METHODS

The techniques of plant extraction include percolation, maceration, digestion, infusion, decoction, hot continuous extraction (Soxhlet), aqueous‐alcoholic extraction by fermentation, counter‐current extraction (CCE), microwave‐assisted extraction (MAE), ultrasound‐assisted extraction (UAE), supercritical fluid extraction (SFE), distillation techniques (water distillation, steam distillation, phytonic extraction by using solvents (hydrofluorocarbon solvents) (Trusheva et al., 2007; Sutar et al., 2010; Bimakr et al., 2011; Patil et al., 2014; Manzoor, Ahmad, et al., 2019; Zia et al., 2021; Manzoor, Ahmad, et al., 2019). Specifications that influence the extract's quality are the part of the plant used, solvent utilized, and procedure used. These variations in methods affect the quality of secondary metabolites composition (Pandey & Tripathi, 2014). The lotus seed components can be extracted by various methods are given in Table 3.

**TABLE 3:** Extraction methods for lotus seeds components

Health Benefits	Seed Form	Compounds	Method	Conditions	Target	Reference
Anti‐obesity	Freeze dried lotus seeds	Phenolics	Ethanol extraction (v/v)	Room temperature for 3.7 hr centrifugation (10,000 r/min, 10 min)	3T3‐L1 pre‐adipocytes	(Lin et al., 2019)
	Frozen lotus seeds	Phenolics and flavanoids	Ethanolic extraction	50°C in a water bath for 2 hr	Adipocytes	(You et al., 2014)
Anti‐inflammatory	Lotus seeds	Phenolics	Hexane (20%,w/v) Ethanol 70%	At room temperature for 2 hr	LPS‐stimulated RAW264.7 macrophages	(Moon et al., 2019)
	Powders of lotus plumule	Flavonoids	Ultrasound assisted extraction (70% ethanol)	50 min, 50°C and ultrasonic power level 100	Inflammatory mediators (PGE_2_, NO, TNFα) and pro‐inflammatory cytokines (IL−6, IL−1β)	(Chen, Fan, et al., 2019)
Immunomodulatory	Rhizome and seeds	‐	Ethanolic extraction (70% ethanol by cold maceration process)	Rotary evaporator used for evaporation of extracts in a period of 15 days	Immune system	(Kumar et al., 2011; Mukherjee et al., 2010)
	*N*. *nucifera* seeds	Betulinic acid and a steroidal pentacyclic triterpenoid	Ethanolic extraction (*n*‐hexane, ethyl acetate, butanol)	Stored at 4°C	Peripheral blood mononuclear cells	(Liu et al., 2004)
Hepatoprotective	Lotus seeds germ oil	Phenolic compound, carotene and tocopherols	Supercritical fluid extraction	0.5 L/min, CO2 flow rate, 2hr time, 32MPa pressure, 45°C	Liver damage	(Lv et al., 2012)
	Lotus seeds plumule	Liensine, Isoliensinie, Neferine	UAE	220 W ultrasound at 30°C for 45 min		(Liu et al., 2019)
	Lotus seed pods	Flavonoids	Aqueous extract	Macerated with hot water at 95°C then filtered under vacuum at −85°C then lyophilized and stored at −20°C		(Tseng et al., 2019)
Anti‐hypertensive	Lotus plumule	Alkaloids	Ethanolic extraction (50%–95%)	Place in a water bath for 1–3 hr, used rotary evaporator under reduced pressure for concentration	Aorta	(Etsassala et al., 2019)
Antioxidant activity	Lotus seeds	‐	Solvent extraction by (100 ml hexane)	Soxhlet method	Macrophage RAW 264.7 cells	(Li et al., 2009; Xie et al., 2013; Yen et al., 2006)
	Lotus plumule	Alkaloids	Solvent extraction (80% ethanol)	After cooling at room temperature, extract centrifuged at 1,500 g for 15 min	Oxidative stress	
	Essential oils from lotus plumule	Betulinic acid and steroidal pentacyclic triterpenoid	The supercritical CO_2_ extraction procedure	0.5 L/min CO_2_ flow rate, 2 hr time, 32 MPa extraction pressure, 50°C extraction temperature, 9 MPa separate pressure and 45°C separate temperature.	Inhibitory effect on free radicals	
Anti‐cancer	*Nelumbo nucifera* seeds	Alkaloids	Methanol extraction	Dried in vacuum at 40°C for 3 hr, Repeated chromatography of the CH_2_Cl_2_ fraction over a Si gel column with benzene–EtOAc–diethylamine afforded the neferine	HEp3B cells	(Yoon et al., 2013)
	Lotus seeds	Alkaloids	Solvent extraction (1,000 ml of Ethanol 80% vol/vol)	At 50°C for 1 hr, Following filtering, the extraction solution was loaded into an 80 cm cation exchange resin 732 columns at 50°C and the filtrate collected 3 hr later. In the end, ethanol eluent finely condensed using a vacuum rotary evaporator at 37°C, then freeze‐dried	Nasopharyngeal cells	(Zhao et al., 2016)
	Lotus seeds	‐	Solvent extraction method	1,000 ml ethanol (70%, v/v) at 50°C for 1 hr. After filtering, the sample extraction solution was condensed by a vacuum rotary evaporator	Human colon cancer cell HCT−116 cells	(Zhao et al., 2017)
Anti‐viral	Lotus seeds	‐	Solvent extraction	After removing the solvent, the crude extracts were dissolved in dimethyl sulfoxide (DMSO) to a concentration of 100 mg/ml and stored at 4°C until further use	HeLa cells	(Kuo et al., 2005)
Anti‐diarrhoeal	Oil from seeds	Flavonoids	Soxhlet's procedure	The seed was oven‐dried at 105°C, then washing is done in Soxhlet with Hexane (boiling point 40 to 60°C). the solvent evaporated under a vacuum in a rotary evaporator.	Salmonella sp., Klebsiella sp., *S*. *aureus*, *E. coli,* Peudomonas sp., and Shigella	(Arumugam & Dhailappan, 2012)
Anti‐microbial	Lotus seed pods	Procyanidins	Solvent extraction	70% ethanol at 50°C for 1.5 hr. The crude procyanidin aqueous solution was loaded onto an AB−8 resin column, then evaporated to get procyanidin	Strains of *E. coli*,	(Tang et al., 2017)
Anti‐fertility	Lotus seeds	‐	Soxhlet's extraction	50% ethanol	Female reproductive system	(Mutreja et al., 2008)
	Lotus seeds	‐	Solvent extraction	Seeds dried in a hot air oven at 60°C for 7 days, Dried seeds (50g) macerated with 75% ethanol (500 ml) for 14 days. The extract filtered through Whatman paper No. 1 and dried by a rotary evaporator	Sexual behavior	Wethangkaboworn & Munglue, 2014)
Anti‐depressant	Lotus seed embryo	Alkaloids	Solvent extraction	Hot Methanol used, for evaporation vacuum used, *n*‐hexane and BuOH	Depression	(Sugimoto et al., 2008)
Analgesic effect	Red and white lotus seeds	Phenolics	Methanolic extract	Soxhlet apparatus	Pain and inflammation	(Chakravarthi et al., 2009)
	Lotus seeds powder	‐	Cold extraction (ethanol 95%)	Condensed pressure 40 to 45°C then freeze‐dried at −30°C		(Rajput et al., 2019)
Anti‐aging effect	Lotus seeds	‐	Freeze‐dried water extraction (twice)	Heat at 100°C until the volume is 50% then cooled at RT then preserved with 20% propylene glycol	Wrinkles	(Kim et al., 2011)
Anti‐diabetic effect	Lotus seeds resistant starch		Ultrasonic and autoclaving	Ultrasonic power of 300 W for 55 min at 25°C then pressure‐cooked in an autoclave at 115°C for 15 min, cooled to RT, and stored at 4°C for 24 hr. Finally, dried at 50°C, grounded using a ring sieve with an aperture size of 185 μm and then purified	Diabetes	(Wang et al., 2018; Zeng et al., 2015)

## 6 THERAPEUTIC POTENTIALS

The market of nutraceuticals and functional foods has existed for many years but it is very difficult to predict the future because of provocations such as country rules, problems in sustainability of health claims, and non‐innovative behavior of food industries (El Sohaimy, 2012). Globally, the acceptance for functional food is mixed. Although many countries have rules for the regulation of health claims, still this has not enough for the application of these claims practically. The therapeutic and nutraceuticals foods with special ingredients may be patent yet many of these products have free ingredients and can easily be copied. These circumstances provide somewhat benefit to the initiating companies. Additionally, these nutraceuticals and functional foods address the health claims and health benefits, so these claims must be proven scientifically, with evidence‐based researches. Not all this is as simple as it appears because the biological markers related to health improvement or disease reduction are difficult to recognize. Moreover, clinical trials took a long time and the effective dosage along with their adverse effect usually require supplemental research (Daliri & Lee, 2015). The therapeutic foods are usually prepared for a targeted population that is, specific disease or at risk of that disease such as CVDs, diabetes, obesity, and allergy. Globally, all the species of lotus seeds are widely cultivated as they have several health benefits. These seeds have been known and cultivated since ancient times. Besides, the therapeutic and functional properties of lotus seeds have been a source of great interest toward the pharmaceutical industries and nutritionists as the seeds have a wide variety of bioactive constituents, such as vitamins, minerals, and phenolic compounds. These bioactive components impart many health benefits to humans (Altemimi et al., 2017).

### 6.1 Anti‐adipogenic effect

**FIGURE 1:**
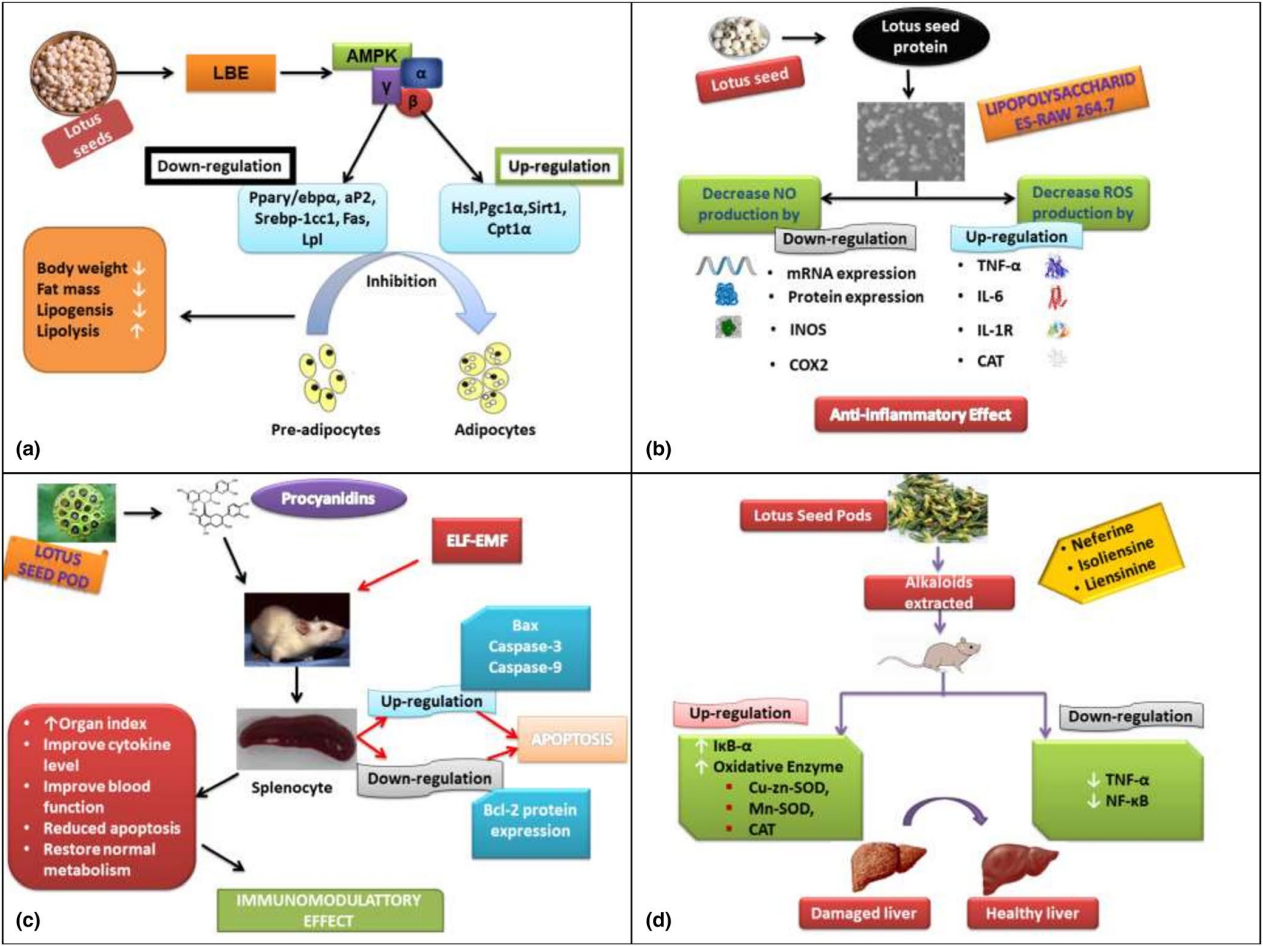
(a) shows that Lotus Bound Phenolic (LBP) significantly lowered the intracellular accumulation of lipid (b) shows the anti‐inflammatory effect of lotus seed protein in RAW 264.7 macrophages (c) shows that the LPSCs (lotus seed procyanidins) by ELF‐EMF (extremely low frequency electromagnetic field) exposure and their shielding mechanism opposite to harmful radiation (d) shows that the alkaloid extract of lotus plumule was used against liver injuries induced by carbon tetra chloride

Obesity is a complex disease that affects most of the world's population, and it has been linked with various diseases including hypertension, diabetes mellitus, and cardiovascular disease. Thus, it is of great significance to identify food that reduces obesity (Achike et al., 2011). Lotus seeds extract inhibited the process of adipogenesis and it reduced the weight of adipose tissues, improved blood lipid profile, and attenuated level of serum leptin in rat study (Joint & Organization, 1973). Another study was conducted to explore the anti‐obesity effect of the bound phenolic component present in lotus seed in 3T3‐L1 pre‐adipocytes cells of mice. Moreover, Lotus Bound Phenolic (LBP) significantly lowered the intracellular accumulation of lipid while LBP delayed the weight gain and ameliorated plasma lipid profile in high fat‐induced mice. Also, phenolic increased the phosphorylation of adenosine mono‐phosphate‐activated protein kinase (AMPK). It also downregulated the gene (Ppary/ebpα, Srebp‐1cc1, aP2, Fas, Lpl) expression, upregulation of lipolytic genes (Hsl, Pgc‐1α, Sirt1, Cpt1α), and adipokine adiponectin expression as shown in Figure 1(a). Phenolic present in lotus seed plays an important role in ameliorating the AMPK signaling pathway. Adenosine monophosphate‐activated protein kinase plays an important role as a metabolic sensor and also regulates adipogenesis (Lin et al., 2019).

### 6.2 Anti‐inflammatory activity

Inflammation is a defensive response of living organisms to external or injurious stimuli. This process eliminates exogenous stimuli and damaged tissue followed by initiating tissue repair. The inflammatory response involves many immune cells, such as neutrophils, mononuclear phagocytes, and macrophages. This response release inflammatory mediators (TNF‐α, NO, IL), inflammatory proteins (COX‐2), and inducible nitric oxide synthase upon stimulation by exogenous stimuli such as lipopolysaccharide (LPS) in Gram‐negative bacteria (Abarikwu, 2015; Ahn et al., 2016; Reddy & Reddanna, 2009;). The anti‐inflammatory effect of lotus seed protein on LPS stimulated RAW 264.7 macrophages was investigated. It has been found that stimulated RAW 264.7 macrophages after lotus seedpod isolate (LSPI) treatment resulted in a decreased NO production by downregulation of protein and messenger RNA. It also attenuated the production of reactive oxygen species (ROS) by upregulation of TNF‐α, catalase activity, interleukin‐6, and IL‐β in LPS stimulated RAW 264.7 macrophages as shown in Figure 1(b) (Moon et al., 2019). The anti‐inflammatory action of flavonoids from the plumule of the lotus was determined. In this experiment, flavonoids showed an anti‐inflammatory effect by reducing the production of the inflammatory mediators (PGE_2_, NO, TNFα) and pro‐inflammatory cytokines (IL‐6, IL‐1β) (Chen, Fan, et al., 2019).

### 6.3 Immunomodulatory activity

The immune system is a body's defense mechanism that protects the body from harmful pathogens. It produces an immediate response by using specific receptors that activate immune cells, chemokine, cytokines and release inflammatory mediators. The lotus plant contains betulinic acid and a steroidal pentacyclic triterpenoid, these plant extracts are used for the immunomodulatory activity (Kumar et al., 2011). Hydroalcoholic extract of rhizomes and seeds of *N. nucifera* was identified and it showed that the extract of *N. nucifera* has a stimulating effect on the immune system by ameliorating the parameters of the immune system, and the parts of the plant have therapeutic potentials on the immune system (Mukherjee et al., 2010). Lotus ethyl alcohol extracts effect on peripheral blood mononuclear cells (PBMC) was investigated. It stimulated phytohemagglutinin to hinder cytokine production and cell proliferation (Liu et al., 2004). Another study explored that (S)‐armepavine (C_19_H_23_O_3_N; MW313) extracted from lotus seeds suppressed T cells proliferation and presented its therapeutic potentials including immune diseases, systemic lupus erythematosus, this potential was examined on MRL/MpJ/1‐pr mice as an in vivo model having disease characteristics similar to human systemic lupus erythematosus. Results have shown that (S)‐Armepavine prevented lymphadenopathy and extended the mice life span. It also significantly decreased the T lymphocyte‐mediated cytokine production (Liu, Tsai, et al., 2006). Zhang et al., (2016) investigated the protective effect of lotus seeds on mice organs damaged by exposure to ELF‐EMF radiations. While results showed that lotus seed procyanidins enhanced the organ index of mice and cytokine levels by extremely low‐frequency electromagnetic field radiation recuperated to normal appearance. Moreover, this experiment proved that the dosing of lotus seedpod proanthocyanidins (LSPC) restored normal cell metabolism and reduced the apoptosis of spleen cells. Moreover, LSPCs prevented the reduction in DNA content that was caused by ELF‐EMF. Western blot estimated the levels of genes that caused apoptosis including Bcl‐2, Bax, Bcl‐cl, Caspase‐3, and Caspase‐9. Furthermore, significant suppression in Bcl‐2 expression and elevation in Bax, Caspase‐9, and Caspase‐3 expression in splenic cells in ELF‐EMF group as shown in Figure 1(c). However, LSPCs recovered these changes (Zhang et al., 2016).

### 6.4 Hepato‐protective effect

The liver is present in the abdominal cavity and the largest organ playing an essential physiological function in human beings. Blood is abundantly supplied through hepatic arteries and portal veins, since hepatocytes are vulnerable to hypoxia, therefore, blood should be supplied properly. Hepatic injury if not treated may threaten the health of the human and can even cause death (Stawicki, 2017). Nowadays degree of liver damage is assessed clinically with various indicators that include a liver index, liver tissue oxidation, and serum biochemical index (Wang et al., 2009). An experiment was evaluated to investigate the defensive effect of lotus seeds on kidney and liver tissue damage induced by CCl_4_ in mice. It illustrated the anti‐oxidative power of lotus seeds by protecting the tissues from oxidation (Lv et al., 2012). Another research was conducted in which alkaloids extract of lotus plumule was used against liver injuries, induced by carbon tetrachloride. The experiment disclosed the hepato‐protective effect by upregulating the expression of IκB‐α (inhibitor of NF‐κB alpha), messenger RNA (mRNA), catalase (CAT), copper/zinc superoxide dismutase (Cu/Zn‐SOD), manganese superoxide dismutase (Mn‐SOD), downregulating the expression of nuclear factor kappa B (NF‐κB) and the tumor necrosis factor‐alpha (TNF‐α) as shown in Figure 1(d) (Liu et al., 2019). A flavonoid‐rich extract of lotus seed pods exhibited hepato‐protective effect against hepatic inflammation induced by LPS in mice by inhibiting the expression of pro‐inflammatory cytokines and mediators (Tseng et al., 2019).

### 6.5 Anti‐Alzheimer activity

**FIGURE 2:**
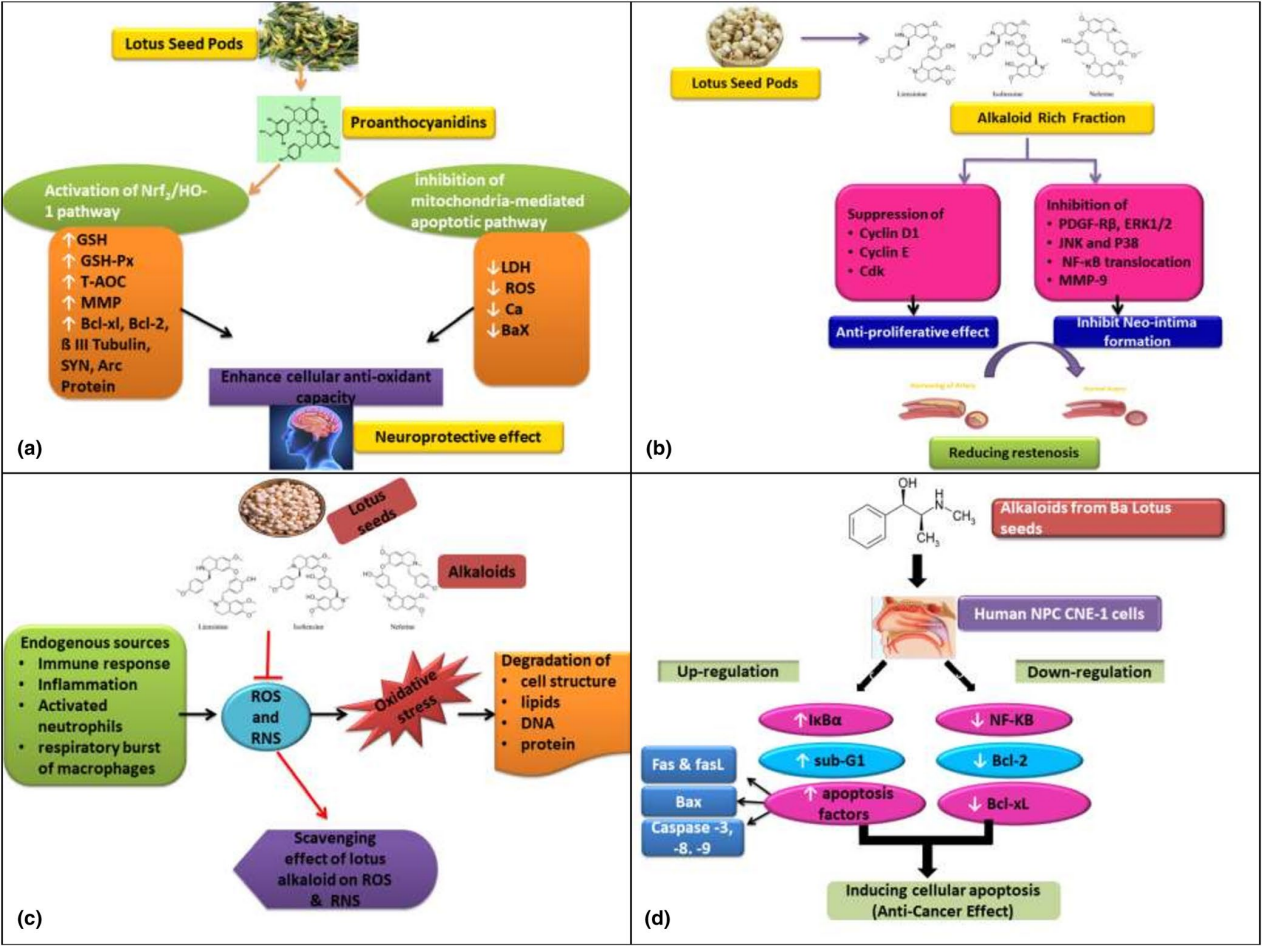
(a) shows the mechanism on how lotus seedpod proanthcyanidins (LSPC) prevented neurotoxicity (b) inhibitory effect of lotus seeds alkaloid rich extract on neo‐intima formation and VSMC proliferation in a rat model (c) shows that the total crude alkaloids and three main alkaloids such as liensinine, roemerine and neferine manifest strong protective effect on oxidative stress (d) shows that alkaloid extracted from Ba lotus seeds were used as an anti‐cancerous agent in nasopharyngeal carcinoma oh human CNE‐1 cells

Alzheimer's disease (AD) is a neurodegenerative disorder due to memory loss and other mental impairments. The causes of AD are amyloid plaque, neural cell death, and neurofibrillary tangles (Swerdlow, 2007). The neuroprotective effect of the embryo of lotus seeds was analyzed in HT22 cells through glutamate‐induced cytotoxicity. Seeds were given to mice in different amounts, which had ameliorated the memory impairment and inhibited the activity of acetyl cholinesterase. It also showed a neuroprotective effect through a decrease in ROS level and intracellular accumulation of calcium, thereby treating and preventing AD (Kim et al., 2014). The lotus seedpod proanthocyanidins LSPC have an ameliorative effect on brain aging and cognitive impairment induced by using D‐galactose. Three different doses of LSPC were given to mice after disease induction. The LSPC reduced the level of nitric oxide, malondialdehyde, and β‐amyloid and increased SOD and peroxidase resulting neuroprotective effect. LSPC reduced the expression of P35 protein in the brain ultimately treating AD (Gong et al., 2016). The mechanism of how LSPC prevented neurotoxicity was investigated. Results showed that LSPC up‐regulated the expression of certain transcriptional factors with the increase in dosage. It reduced the expression of Bax protein and increased the expression of Arc, SYN, Bcl‐xl, Bcl‐2 protein as shown in Figure 2(a). The study suggested that lotus seeds enhanced the antioxidant activity through activation of Nrf2/HO‐1 and inhibiting apoptotic signaling pathway (Zhang et al., 2019).

### 6.6 Reducing restenosis and atherosclerosis

Atherosclerosis is characterized by an abnormality in vascular smooth muscle cell (VSMC) migration and proliferation that ultimately causes plague formation whereas restenosis is a recurrence of stenosis (Bennett et al., 2016). A study conducted to evaluate the inhibitory effect of lotus seeds alkaloid rich extract on neo‐intima formation and VSMC proliferation in a rat model. Results showed that extract possessed the strongest antioxidants and anti‐proliferative activity due to suppression of cyclin D1, cyclin E, and cyclin‐dependent kinase (Cdk) gene expression and inhibition of PDGF‐Rb mediated signaling as shown in Figure 2(b) (Jun et al., 2016). The protective effect of alkaloid from lotus seeds on the aorta during hypertension investigated on male rats. The rats were given the extract for almost eight weeks. This treatment in a dose‐dependent manner reduced the expression of protein collagen I induced by angiotensin II and elevated α‐SMA in the aorta, thereby normalizing the effect of hypertension. Moreover, the regulation of the pathway of RhoA/ROCK and remodeling of vascular smooth muscle cytoskeleton repression caused by the seeds are also responsible for the anti‐hypertensive effect and aortic protective effect (Etsassala et al., 2019).

### 6.7 Antioxidant

The protective and scavenging effects of lotus seed extract against the reactive nitrogen including peroxynitrite‐induced cytotoxicity and sodium nitroprusside, damage of DNA in macrophages RAW 264.7 cells were explored. Lotus seeds extract scavenged the reactive nitrogen species and NO accumulation and acted as chemo‐preventers by the depletion of the surplus quantity of nitric oxide (Yen et al., 2006). Moreover, lotus plumules essential oil extracted by supercritical fluid extraction also revealed an inhibitory effect on hydroxyl (OH) and O_2_‐free radicals in a dose‐dependent manner (Li et al., 2009). The lotus epicarps also possess strong antioxidant potential containing a large number of flavonoids (Chen et al., 2012; Kredy et al., 2010; Liu et al., 2015). Furthermore, the total crude alkaloids and three main alkaloids such as liensinine, roemerine, and neferine manifested the strongest protective effect on oxidative stress induced by tert‐butyl hydroperoxide in the human hepatocellular HepG2 cell line. This shielding effect was linked with the decrease in the formation of reactive oxygen species as shown in Figure 2(c), thiobarbituric acid‐reactive substance generation, lactate dehydrogenase release, and increases in GSH levels in a dose‐dependent manner, suggesting the involvement of alkaloids in the cytoprotective effects against oxidative stress (Xie et al., 2013).

### 6.8 Anti‐cancer

Cancer is the second principal cause of death and the major health issue worldwide. Cancers are carcinomas and sarcomas that originate from uncontrollable and abnormal cell division that destroy the cells and tissues surrounding them (Shipitsin & Polyak, 2008). The anti‐cancer effect of neferine, an alkaloid isolated from *N. nucifera* seeds on Hep3B cells by inducing cell cycle arrest was investigated. The result manifested that the alkaloid presents not only induced apoptosis but also the endoplasmic reticulum (ER) stress by the activation of caspases, Puma, Bak, Bax, Bim, Bid, and upregulation of certain proteins (Yoon et al., 2013). The alkaloids extracted from Ba lotus seeds were used as an anti‐cancer agent in nasopharyngeal carcinoma on human CNE‐1 cells. This lotus seeds extract reduced the proliferation of cells in a dose‐dependent manner. The results showed an increase in apoptosis‐related factors such as caspase family, Bcl‐2 associated X protein, Fas and Fas ligand with the decrease in anti‐apoptotic proteins expression as shown in Figure 2(d) (Zhao et al., 2016). An experiment was done on the ethanolic extract of lotus seeds against carcinoma of the human colon HCT 116 cell line, which induced the anti‐cancer effect by increasing the number of apoptotic factors and thereby decreasing the protein expression of anti‐apoptotic factors (Zhao et al., 2017).

### 6.9 Antithrombotic activity

**FIGURE 3:**
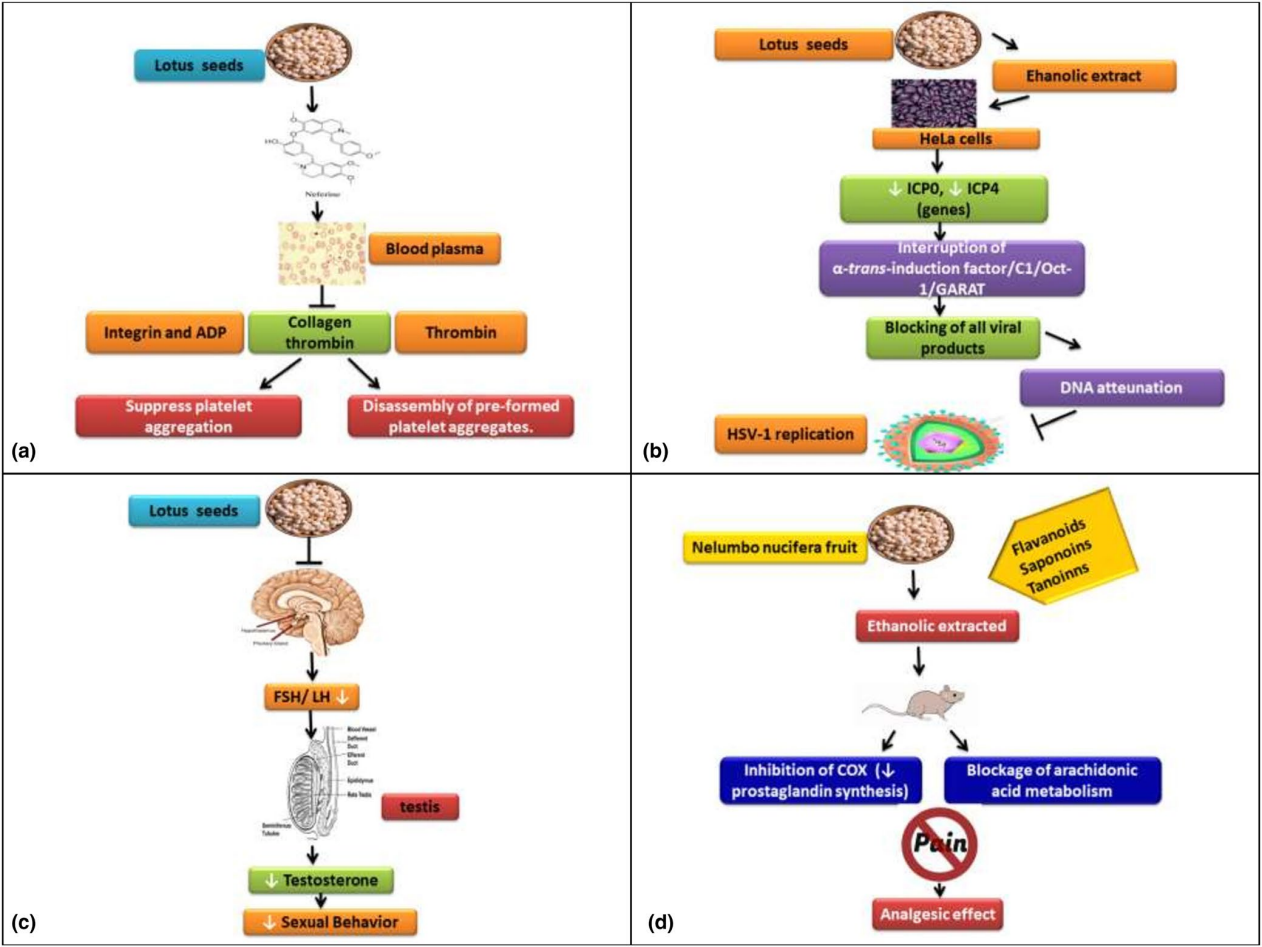
(a) shows the antithrombotic effect by inhibiting platelet aggregation and promoting dissociation of platelet aggregates (b) shows the effect of lotus seed extract restrain the herpes simplex type‐1 replication, (c) shows the effect of extract of lotus seed on male rat sexual behavior, (d) the effect of lotus seeds against pain and inflammation in rat model

Platelets are very small cells without a nucleus that plays a very important role in the homeostatic process. When blood vessels are injured due to some reason then platelets are activated by the interaction of receptors that are present on the cell surface with collagen (Bye et al., 2016). This interaction of receptors and collagen results in subsequent activation of platelets by soluble agonists like adenosine diphosphate, thromoboxane A2, and thrombin that leads to cascade formation of intracellular events which includes movement of calcium, secretion of granule and activation of integrin. This results in the accumulation of platelet, development of thrombi, and the stoppage of bleeding (Nieswandt et al., 2011). A study by Zhou et al. (2013) stated that neferine lessens the platelet dense‐granule secretion that is induced by collagen and thrombin, U46619. It hindered the U46619, thrombin, and collagen‐induced platelet aggregation in mice washed‐platelets or platelet aggregation induced by ADP in plasma rich protein (PRP) of mice. Neferine also upgraded the splitting of platelet aggregates, which were pre‐formed by different agonists including thrombin, U46619, collagen, or ADP, thus specifying, its anti‐thrombotic activity as shown in Figure 3(a). Meantime, neferine might also remarkably prolonged carotid‐occlusive thrombosis time in rats by electrical stimulation (Zhou et al., 2013).

### 6.10 Anti‐viral activity

Lotus seeds extract restrain the herpes simplex (type‐1) virus replication using IC50 for replication. The sub‐fractions NN‐B (*N. nucifera* Butanol) were separated from the seeds. Out of nine main fractions, NN‐B‐1 to NN‐B‐9, NN‐B‐5 had the highest inhibitory effect on herpes simplex virus‐1. Results of polymerase chain reaction and Southern blotting revealed that HeLa cells treated with NN‐B‐5 have impaired DNA replication, mRNA transcription of infected cell protein (ICP)_0_ and ICP_4_ were lowered in NN‐B‐5 treated HeLa cells in herpes simplex viruses as shown in Figure 3(b). The NN‐B‐5, at 50 μg/ml had suppressed HSV‐1 replication within HeLa cells equivalent to 85.9%. The results of the study suggested evidently that NN‐B‐5 weakened the propagation of acyclovir‐resistant HSV‐1 (Kuo et al., 2005).

### 6.11 Anti‐microbial activity

Lotus seeds oil was used for the anti‐diarrhoeal effect and it inhibited the microbial strain including Salmonella sp., Klebsiella sp., *Staphylococcus aureus*, *Escherichia coli*. Pseudomonas sp. and Shigella. The anti‐microbial activity was investigated by the use of the disk diffusion method. The pathogenic activity of dermatophytes (*Trichophyton rubrum*, *Trichophyton mentagrophytes,* and *Malassezia furfur*) was suppressed by using 25 µg/ml concentration of lotus seeds oil against it (Arumugam & Dhailappan, 2012). The anti‐microbial activity of procyanidins from pods of lotus seeds against two strains of *E*. *coli* as well as on the growth of six helpful bacteria was investigated. In a dose‐dependent manner, pods of lotus seeds exert their effect on *E*. *coli* strains in which growth was promoted at low concentration but at a concentration greater than 1.2 mg/ml growth was inhibited. In addition, the concentration of 0.8 mg/ml pods of lotus seeds promoted the growth of Lactobacillus strains. Lactobacillus strains are generally involved in regulating gut microbiota (Tang et al., 2017). Hence, LSPC showed a beneficial effect in microbial growth regulation by selectively suppressing the Enterotoxigenic *Escherichia coli* (ETEC) strains growth but promoting lactobacillus growth at a specific concentration range. The cell morphology, cell integrity, and cell permeability assays proposed that the mechanism of pods of lotus seeds against ETEC strains was due to disrupting structure as well as the function of the plasma membrane. The result of this study was elaborating the potential effect of pods of lotus seeds on gut microbial regulation (Tang et al., 2017).

### 6.12 Anti‐fertility activity

Studies have demonstrated that ethanolic extract of lotus seeds have a positive effect on the female reproductive system at a dose of 800 mg/kg for 40 days. Seeds significantly lowered the weight of the ovary, vagina, and uterus as well as glycogen and protein level in these organs. Thus, the seed extract has anti‐estrogenic nature without changing the general physiology of female rats (Mutreja et al., 2008). The ethanolic extract of lotus seeds affects the reproductive function and fertility of male rats. The extract at a dose level of 50, 100, and 200 mg/rat/day was given to male rats orally for 60 days and found that the weight of the body was not changed but after this treatment weight of reproductive organs was decreased. It also suppressed the cauda epididymal sperm count and motility. The testicular concentration of cholesterol was remarkably raised, whereas sialic acid, fructose, glycogen, and protein content were decreased significantly. Through this study, in male rats, lotus seeds have an anti‐spermatogenic effect (Chauhan et al., 2009). In another study, lotus seeds extract was used on male rat sexual behavior and compared with the standard reference drug, sildenafil citrate. The effect of ethanolic seed extract of *N. nucifera* on male rat sexual behavior as presented in Figure 3(c). The extract had significantly increased the Mounting Latency, Intromission Latency, and caused a significant decrease in Ejaculatory Frequency in Second Series (Wethangkaboworn & Munglue, 2014).

### 6.13 Analgesic activity

Pain can be considered in two categories viz. nociceptive and neuropathic pain. Nociceptive pain results from unrealized or actual tissue damage. The neuropathic pain occurs due to damage or injury to the peripheral nervous system (PNS) and central nervous system (CNS) and it remains even after all the signs of original injury have disappeared (Wieczorkiewicz‐Plaza et al., 2004). In order to verify the lotus seed's analgesic effect, an experiment was conducted in which rats were divided into six different groups, each having eight rats. Group 1 was taken as control group 2 was given standard analgesic drug (diclofenac) while the other groups were given two different varieties (red and white) of lotus seeds in amount 400 mg/kg and 600 mg/kg for about seven days. After that, counting of foot withdrawal reflex of every group was done using cold stimulus induced by acetone and revealed that 600 mg/kg of body weight of white lotus seeds had the highest analgesic activity (Chakravarthi et al., 2009). Another research was conducted to explore the effect of lotus seeds against pain and inflammation in a rat model. The outcome of the experiment showed that a dosage of 200 mg/kg increased the percentage of tail elongation time and reduced the count of writhes due to the presence of flavonoids and other secondary metabolites which inhibited the arachidonic acid pathway as shown in Figure 3(d) (Rajput et al., 2019).

### 6.14 Anti‐aging

**FIGURE 4:**
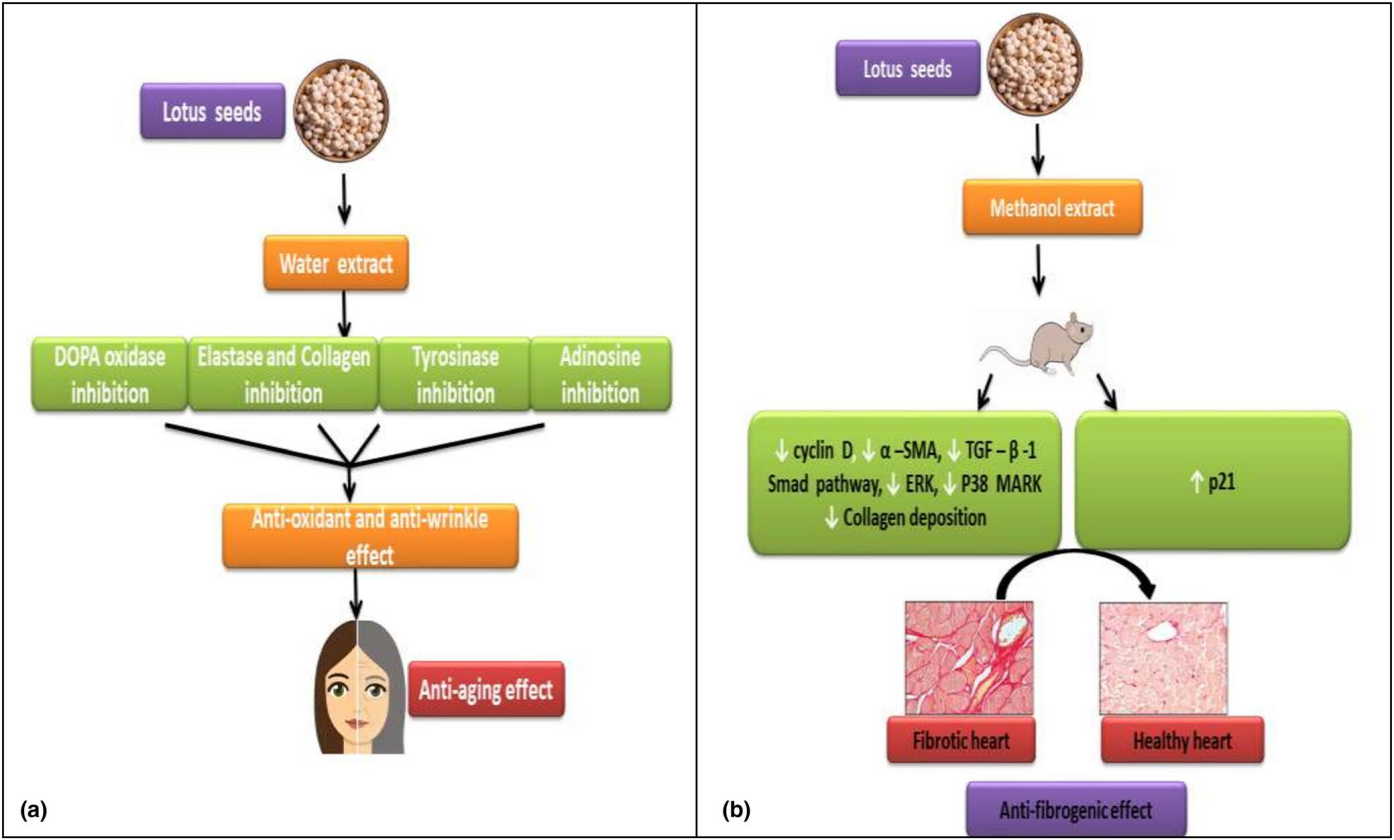
(a) shows the anti‐aging effect of lotus seed water extract. (b) Shows the anti‐fibrogenic effect of lotus seeds in rat model

The process of aging of the skin can be classified into two categories that is, aging due to factors, which are internal such as age, and due to factors, which are external such as sunlight (photo‐aging). Aging due to external factors, specifically due to ultraviolet light (UV) rays, is called photo‐aging (Bissett et al., 1987). UV radiation is the most important environmental factors affecting the health of human being. From the studies mentioned below, it is evident that lotus seeds have also a role in skin protection, care and is widely used as anti‐wrinkle and whitening agent. Kim and Moon (2015) studied the role of seeds in skin protection from sunlight and scars. For this purpose, lotus seed tea (LST ) was used for six months in one of the two groups of the hairless mouse model, after six months with the exposure of ultraviolet rays the LST group showed clear evidence of skin protection (Kim & Moon, 2015). The functional properties of lotus seeds, *Nelumbinis semen,* as a cosmetic agent was tested. The elastase inhibition assay was done which showed that seeds possessed an anti‐wrinkle effect while the tyrosinase inhibition assay and DOPA‐oxidase inhibition assay showed the whitening effect of lotus seeds as shown in Figure 4(a) (Kim et al., 2011).

### 6.15 Anti‐depressant effect

Depression is a common, but very serious illness that devitalizes motivation. Serotonin (5‐HT) and noradrenaline are the monoamines in the brain related to depression. A high level of these monoamines or neurotransmitter in the synapse improves the condition of depression and mostly anti‐depressant also act on these neurotransmitters in the neural system (Trevor et al., 2010). Nowadays anti‐depressants have been used widely to treat depression but due to their excessive use, most patients have become resistant to them and therefore now patients are heading toward traditional plants and medicine of therapeutic potential. A study was conducted to evaluate the anti‐depressant effect of lotus seeds ethanolic extract on mice model. The alkaloids mainly present in lotus seeds induced hypothermia, increased time of sleep, and inhibited the locomotion in mice. The alkaloid neferine also showed an anti‐anxiety effect (Sugimoto et al., 2008). The impact of neferine on immobility was utilized to evaluate its anti‐depressant activity. Neferine was administrated to mice from 25 to 100 mg/kg. Forced swimming test indicated that neferine has the same effect as anti‐depressant medicines and these effects were mediated by 5‐HT_1A_ receptor (Sugimoto et al., 2010). A study was designed for phytochemical screening along with evaluating anxiolytic and anti‐depressant activity of the lotus seeds extract. It showed that the seeds extract was rich in flavonoids, saponins, alkaloids, and tannins. Moreover, lotus seeds are given in an amount of 100 and 200 mg/kg showed significant anti‐anxiety effect as demonstrated through an elevated plus maze and the light and dark test. An increase in time spent in the light observed when compared to control in light–dark test. In addition, a turndown recorded in the duration of immobility on day 15 that is, after administration of dose for 14 days. Thus, lotus seeds manifested significant anti‐depressant and anxiolytic effects, and proved their usage as a therapeutic agent against anxiety and depression (Rajput & Khan, 2017).

### 6.16 Anti‐fibrogenic effect

A change in the cellular composition of the alveoli due to superfluous deposition of collagen is the major characteristic of pulmonary fibrosis (PF) regardless of this, the main cause is unknown. In pulmonary fibro proliferative disorders, the major elementary component is lung inflammation. A study was conducted in which the effect of isoliensinine (IL), an alkaloid extracted from *N. nucifera* Gaertn seeds against pulmonary fibrosis induced in mice. The protective effect was due to increased hydroxyproline and malondialdehyde (MDA) levels with decreased serum superoxide dismutase (SOD) activity in lung tissues and serum (Xiao et al., 2005). The methanol extract of *N. nucifera* seeds inhibited locomotor activity in mice. Moreover, neferine extracted showed an anti‐anxiety effect, hypothermia, anti‐fever effects, and anxiolytic effects comparable with those of diazepam but with a different mechanism (Sugimoto et al., 2008). Cardiac fibroblasts are the cells present in the heart that are mainly responsible for removing and degradation of extracellular fluid in the tissues of the heart. This fibroblast is crucially involved in conditions like cardiac fibrosis as matrix‐producing cells (Souders et al., 2009). In a reported study, the effect of neferine on cardio fibrosis was observed which was induced by diabetes mellitus. This alkaloid reduced left ventricle (LV) dysfunction and collagen deposition as in diabetes. It also prevented the proliferation of fibroblast and its migration, differentiation into myofibroblast through inhibition of TGF‐β1‐Smad, extracellular receptor kinase (ERK), and p38 MAPK signaling activation as shown in Figure 4(b) (Liu et al., 2016).

### 6.17 Hypoglycemic Activity

Studies have demonstrated the hypoglycemic effect of inorganic constituents in lotus seeds on streptozotocin‐induced diabetes in rats. These inorganic constituents involved mainly in hypoglycemia are trace elements, important in biological systems. The trace elements that are present in lotus seeds are chromium, potassium, sodium, calcium, magnesium, and manganese. These minerals might play a direct and indirect role in insulin secretion and maintain a normal level of glucose in the body by this action (Mani et al., 2010). The hypoglycemic activity of lotus seed resistance starch (LSRS) on diabetic mice was determined. LSRS remarkably lowered the level of blood glucose by 16 to 33.6 percent; recuperated the level of serum insulin by 25 to 39 percent in mice. Results showed that the LSRS had a protective effect on modulating the different key factors that were involved in insulin signal transmission, insulin secretion, antioxidant activity, p53 signaling pathways, and cell apoptosis (Wang et al., 2018). Another study explained the health‐promoting and functional benefit associated with anti‐diabetic and anti‐obesity effects of two different percentages of *N. semen* powder in high fat‐induced obese mice. The result showed that the food containing 5% and 10% of NS powder reduced body weight and fat weight. They also did improvement in glucose intolerance, regulated the blood glucose level. It reduced the intraperitoneal glucose tolerance test by 12.5% and 15% respectively. It also increased the expression of PPAR‐γ, GLUT4 protein, and decrease TNF‐α protein expression (Hwang & Lee, 2020).

## 7 CONCLUSIONS AND FURTHER CONSIDERATION

In many countries, lotus seeds are used as food and traditional medicinal purpose. They are edible and used to treat a variety of diseases such as skin diseases, tissue inflammation, and many other diseases. They contain a variety of bioactive compounds like flavonoids, antioxidant, alkaloids, etc., and rich in minerals, protein, and fatty acids. Different researches disclose the therapeutic benefits of these seeds. Phytochemicals present in lotus seeds have provided chemical bases for modern and traditional uses; we intend to cover many compounds from lotus seed reported that is characterized or partially identified by spectroscopic and chromatographic techniques. Moreover, health‐promoting pharmacological and biological activities of lotus seeds extracts and some compounds (like flavonoids and alkaloids) isolated from these extracts, have been described to have positive correlations with those corresponding phytochemicals through numerous in‐vitro and in vivo studies. Furthermore, it is showing less toxicity than that from other synthetic drugs and contains natural compounds. Due to all these properties, lotus seeds have captivated considerable concentration in recent years. For food and pharmaceutical industries, the nutraceutical properties of lotus seed have been of great interest. Importance should be given to the cultivation of lotus on a large scale and processing of seeds so that the general mass of people can consume lotus seeds as a low‐cost nutritious food and use it as a low‐cost medicine for the treatment of diseases. This review highlights several pharmacological and phytochemical studies that have demonstrated the therapeutic potential of lotus seeds. Still, there is a need to work on some potentials to understand their mechanism of action and on clinical studies based on human volunteers to provide evidence‐based therapeutics.

## Data Availability

The dataset supporting the conclusions of this article is included within the article.
